# Beyond the Core-Deficit Hypothesis in Developmental Disorders

**DOI:** 10.1177/0963721420925518

**Published:** 2020-07-15

**Authors:** Duncan E. Astle, Sue Fletcher-Watson

**Affiliations:** 1 MRC Cognition and Brain Sciences Unit, University of Cambridge; 2 Salvesen Mindroom Research Centre, The University of Edinburgh

**Keywords:** cognitive development, developmental psychology, developmental science, developmental disorders

## Abstract

Developmental disorders and childhood learning difficulties encompass complex constellations of relative strengths and weaknesses across multiple aspects of learning, cognition, and behavior. Historically, debate in developmental psychology has been focused largely on the existence and nature of core deficits—the shared mechanistic origin from which all observed profiles within a diagnostic category emerge. The pitfalls of this theoretical approach have been articulated multiple times, but reductionist, core-deficit accounts remain remarkably prevalent. They persist because developmental science still follows the methodological template that accompanies core-deficit theories—highly selective samples, case-control designs, and voxel-wise neuroimaging methods. Fully moving beyond “core-deficit” thinking will require more than identifying its theoretical flaws. It will require a wholesale rethink about the way we design, collect, and analyze developmental data.

Neurodevelopmental diversity results in some children receiving a clinical diagnosis of a developmental disorder and others experiencing learning difficulties in the absence of a diagnosis. As a result, 14% to 30% of children and adolescents worldwide experience barriers to learning (Department for Education, 2018; National Center for Education Statistics, 2020) that vary widely in scope and impact. Some children are formally diagnosed via specialist education services and placed in categories including dyslexia, dyscalculia, or developmental language disorder. Other children, such as those with attention-deficit/hyperactivity disorder (ADHD), dyspraxia, or autism spectrum disorder (hereafter, autism), are normally diagnosed in clinical services. However, many children who struggle will never receive a formal label for their learning difficulty, despite meeting the criteria for multiple different diagnoses (e.g., Bathelt, Holmes, et al., 2018; Holmes, Bryant, the CALM Team, & Gathercole, 2019; Siugzdaite, Bathelt, Holmes, & Astle, 2020; see also Embracing Complexity Coalition, n.d.).

This classification structure has also been used within research to integrate and guide empirical work (Regier, Kuhl, & Kupfer, 2013). As a result, the literature is organized around a patchwork of different theories that provide putative explanations for different recognized disorders. In these theories, a complex array of observable characteristics are frequently categorized according to a single defining neurocognitive deficit. As understanding of a particular set of diagnostic features evolves, most such theories are gradually pruned from the field because of insufficient evidence, counterevidence, or the emergence of better-specified theories. These theoretical accounts fail, but that is their purpose. Choose any clinical categorization of developmental differences, and there is a graveyard of once-popular theoretical accounts, from the magnocellular theory of dyslexia (Stein, 2001) to the mirror-neuron theory of autism (Williams, Whiten, Suddendorf, & Perrett, 2001).

Over and above specific problems with any single theory, this general class of theory is problematic because of the reliance on the very notion of a “core deficit”—something that has been repeatedly debunked both within specific (Happé, Ronald, & Plomin, 2006) and across multiple diagnostic categories (e.g., Pennington, 2006). But in practice, developmental psychologists and developmental-cognitive neuroscientists frequently return to core-deficit theories, either implicitly or explicitly, if not to motivate studies, then to interpret them. Here, we provide a working definition of a core-deficit account, describe the inherent weaknesses in the theory’s accompanying methods and methodology, and argue that building the empirical foundations for more complex (and accurate) theoretical frameworks will require a wholesale rethink in the way we design, collect, and analyze developmental data.

## Identifying a Core-Deficit Hypothesis

A core-deficit hypothesis pins a multiplicity of cognitive, behavioral, and neurobiological phenomena onto a single mechanistic impairment and is assumed to have the power to explain all observed profiles within a particular diagnostic category. To provide one example, in autism, the dominant core-deficit hypothesis since the mid-1980s has been the theory-of-mind model. This model proposes that autistic people uniquely lack the ability to detect, interpret, or understand the mental states of others. Versions of this model vary in the range of behaviors and mental states they attempt to encompass. At its broadest, the theory-of-mind deficit can draw in differences in play, language development, and all types of processing of emotions, including basic emotions, as well as desires, beliefs, and higher-order complex mental states such as suspicion. On a narrower scale, theory-of-mind impairments in autism are understood to apply only to the automatic and easy processing of complex mental states. Until recently, evidence for this latter position has looked fairly robust, but now even this pillar of autism theory is under threat, as innovative research has revealed that the apparent “deficits” in mental-state understanding exhibited by autistic people may apply only to understanding the mental states of nonautistic people (e.g., Crompton et al., 2020). At the same time, there is a growing body of evidence showing that nonautistic people show impairments in detecting and interpreting the mental states of autistic people (e.g., Edey et al., 2016). This reframes the characteristic “deficit” of autism as a typical manifestation of failed communication across different sociocultural groups (Milton, Heasman, & Sheppard, 2018). More importantly for our argument here, the model has never been adequately able to explain the sleep disturbances, sensory sensitivities, intense and consuming interests, or executive difficulties that are equally prevalent among autistic people.

## The Origins of Core-Deficit Theories and Their Persistent Appeal

Across multiple categories, and despite well-articulated perspective articles highlighting their limitations (Happé et al., 2006; Pennington, 2006), core-deficit accounts remain a recurring theme in developmental-disorders research (e.g., Kucian & von Aster, 2015; Mayes, Reilly, & Morgan, 2015). Part of their intuitive appeal is their relative simplicity. Publishing in higher-tier journals is easier when the story is simple, and doing so can quickly turn into a citation gold mine if the field invests in challenging your theory. If a series of findings can be woven together, this provides an ideal way of combining decades of research and cementing its contribution to the field. However, such tapestries are riddled with loose threads that, if pulled, quickly reveal the flaws in their construction.

Examples of these construction flaws are found in the design choices, participant selection, sample sizes, statistical methods, and restrictive range of measures that are hallmarks of core-deficit thinking. These flaws are still prevalent in the wider developmental literature because despite being mindful of the problems inherent in core-deficit theories, we have yet to change the methodological template inherited with them.

### Highly selective samples

Studies of developmental disorders typically use strict exclusion criteria (e.g., Toplak, Jain, & Tannock, 2005; Willcutt et al., 2001), including the removal of children with co-occurring difficulties. But in reality, co-occurring difficulties are the norm rather than the exception (Gillberg, 2010). For example, it is rare to have selective reading or math impairments; the majority of children with one difficulty will also have the other (e.g., Landerl & Moll, 2010). The same is true within clinical samples: 44% of children who receive an ADHD diagnosis would also meet the diagnostic criteria for a learning difficulty, and a similar percentage who meet the latter criteria would also meet the criteria for an ADHD diagnosis (Pastor & Reuben, 2008).

Because most children with the difficulties of interest are excluded, the literature overstates the “purity” of developmental differences. In turn, basing models on highly selective samples biases theory toward simpler core-deficit accounts. Where studies do include children with different diagnoses or with co-occurring difficulties, the purpose is usually to identify what is unique to each diagnosis rather than to establish which dimensions are important for understanding individual outcomes, irrespective of the diagnostic category applied.

### Study designs do not capture heterogeneity

Most studies use a case-control design, with children grouped according to strict inclusion criteria (see above) and then matched to one or more control groups. Significant differences in group-level statistics are then taken as evidence for a specific deficit in the “case” group. Variability in performance within groups is rarely studied, in part because few studies have sufficient power but also because of reliance on univariate statistics. This issue becomes more and more problematic as diagnostic criteria broaden. Regarding heterogeneity as noise to be minimized removes a crucial signal that could lead to a more complex and accurate theoretical conclusion. Although we endorse the application of Occam’s razor to interpretation of findings, we must be wary of parsimony achieved via flawed means.

### Circular logic of measurement selection

Theoretical conclusions about underpinning mechanisms are constrained by our choice of measures. Tasks are often included because they are regarded as the gold standard for distinguishing a particular group from controls, even though the conceptual underpinnings of the task are poorly understood. The logic can become circular: “We always include a theory-of-mind task when studying autistic people, and autism is characterized by theory-of-mind task performance.” The task has become an implicit requirement within the field without any real mechanistic understanding of why different children find it hard. Moreover, the dominance of a specific core-deficit theory with an associated gold-standard measure eliminates consideration of other possibilities: In the case of autism, why not consider an executive-planning or sensory-profiling measure? If the same domains of measurement are selected in every study, to the exclusion of alternatives, then the supposed cardinality of this profile will inevitably be reinforced. But little has been explained, and alternative possible profiles are not documented.

### Neuroimaging methods inherited from the adult literature

Finding a shared neural substrate that purportedly causes the difference is typically taken as strong evidence for a core-deficit theory. But this is largely an artifact of the analytical approach we take to neuroimaging. Most canonical neuroimaging methods assume a direct correspondence between spatially overlapping brain differences (structural or functional) and surface-level cognitive profiles. A voxel-wise approach to analysis will always produce peaks. Despite its dominance, this approach yields remarkably inconsistent results. For example, ADHD has been associated with differences in gray matter within the anterior cingulate cortex (Amico, Stauber, Koutsouleris, & Frodl, 2011), caudate nucleus (Onnink et al., 2014), pallidum (Frodl & Skokauskas, 2012), striatum (Greven et al., 2015), cerebellum (Mackie et al., 2007), prefrontal cortex (Dirlikov et al., 2015), premotor cortex (Mahone et al., 2011), and most parts of the parietal lobe (Shaw et al., 2006).

Our contention is that the assumption of spatial correspondence is not valid for understanding brain–cognition or brain–behavior links in childhood, especially in children with developmental disorders (see also Johnson, 2011). Difficulties that emerge over developmental time can be arrived at via multiple different underlying neural routes—a phenomenon called *equifinality* (Bishop, 1997). There may also be multifinality: The same apparent underlying neural effects can have different consequences for behavior and cognition across children (Siugzdaite et al., 2020). The canonical voxel-wise neuroimaging methods that dominate developmental cognitive neuroscience instead create the false impression that the neural underpinnings of developmental differences are akin to those resulting from acquired focal brain damage, and in turn this implicitly leads us back to “core deficits” in our theoretical interpretation.

## Moving Beyond the Core-Deficit Hypothesis

There is no shortcut to a comprehensive empirical basis for more complex theory. Nonetheless, in this section, we make some nonprescriptive suggestions as a position from which to move forward.

### Well-powered studies with inclusive samples

If our samples are more reflective of the children we are seeking to understand, then our theories, though necessarily more complex, will likely have greater practical value. It is necessary to include participants with different or multiple diagnoses. Emerging first in adult psychiatry (Cuthbert & Insel, 2013; Morris & Cuthbert, 2012), transdiagnostic approaches focus on identifying underlying symptom dimensions that likely span multiple supposed categories (e.g., Astle, Bathelt, The CALM Team, & Holmes, 2019; Bathelt, Holmes, et al., 2018; Bryant, Guy, The CALM Team, & Holmes, 2020; Hawkins, Gathercole, Astle, The CALM Team, & Holmes, 2016; Holmes et al., 2019; Siugzdaite et al., 2020). Within developmental science there are good examples of researchers cutting across hitherto unquestioned diagnostic boundaries in order to identify cognitive symptoms that underpin learning, but they remain relatively rare (e.g., Astle et al., 2019; Casey, Oliveri, & Insel, 2014; Hulme & Snowling, 2009; Peng & Fuchs, 2016; Sonuga-Barke & Coghill, 2014; Zhao & Castellanos, 2016). A review of a transdiagnostic approach is well beyond the scope of the current article, but suffice it to say, contemporary developmental science needs larger and more diverse samples.

### Embracing methods that capture complexity

There is now a growing array of analysis methods allowing researchers to move beyond univariate group comparisons or pairwise associations between variables. A well-developed tool is structural equation modeling (SEM; e.g., Kline, 2015). SEM combines the strengths of path-modeling and latent-variable approaches to allow researchers, for instance, to specify how latent factors may explain the continuous variability on a set of observed measures (e.g., task performance) and the potential causes and consequences of such factors. SEM offers the tools to identify continuous dimensions that cut across diagnostic boundaries or to directly compare competing causal accounts. More advanced variants of SEM, such as latent-growth-curve models and growth-mixture models, can address more sophisticated questions about how changes in one latent construct relate to changes in another (e.g., Hjetland et al., 2019; Kievit, Hofman, & Nation, 2019). Other variants—for example, hierarchical mimic modeling—can be used to identify multiple routes to particular outcomes (equifinality), such as the role that multiple different brain structures might play in developmental changes to a particular aspect of cognition (e.g., Kievit et al., 2016; Ritchie et al., 2015).

Whereas SEM methods are ideal for testing complex theories or pitting theories against one another, other methods are able to handle complexity in a different way. A more recent development from data science that aims to capture complex interrelationships in an exploratory way is network analysis (e.g., Bathelt, Holmes, et al., 2018; Epskamp, Borsboom, & Fried, 2018; Mareva, the CALM team, & Holmes, 2019). The resulting network can open the door to a new toolbox of analytical techniques, such as graph theory. Rather than inferring the presence of singular latent factors, these approaches capture various different ways in which individual measures can be related over developmental time. For example, does phonological processing act like a hub for verbal short-term memory and literacy development, or is there a dynamic relationship between these constructs over time? Network analysis can also capture heterogeneity within a sample and provide a relatively theory-free means of exploring the underlying structure and composition of a data set. With a network analysis, it is possible to identify subgroups of individuals within which the task interrelationships are different, and a strong advantage of this approach is that a network analysis incorporates metrics for identifying these clusters (e.g., Bathelt, Holmes, et al., 2018).

**Fig. 1.**
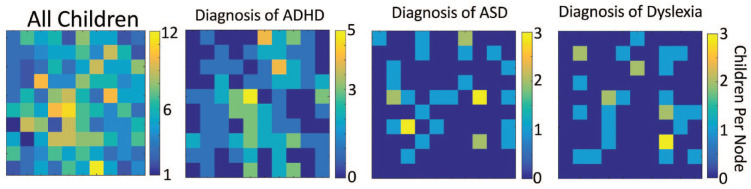
Distributions of children within a simple artificial neural network trained on data from 530 children taken from the Centre for Attention, Learning and Memory (CALM) sample (Holmes, Bryant, the CALM Team, & Gathercole, 2019). Each node represents a profile learned by the algorithm, with spatially nearby nodes having more similar profiles. Therefore, the maps represent the multidimensional spaces that reflect the performance differences across the children. The left-most panel shows the best-matching unit for all children, and the subsequent panels show those for children with different diagnoses. The training data set included measures of fluid reasoning, vocabulary, verbal and spatial short-term and working memory, and phonological awareness (see also Astle, Bathelt, The CALM Team, & Holmes, 2019). ADHD = attention-deficit/hyperactivity disorder; ASD = autism spectrum disorder.

Unsupervised machine-learning techniques are also capable of capturing the multidimensional space in which children may differ (e.g., Siugzdaite et al., 2020). But relative to SEM-based approaches and network analysis, machine-learning applications remain underdeveloped. Although popular in other areas of science with similar challenges, these methods have yet to gain much traction within the study of developmental differences (but see Astle et al., 2019). These algorithms are highly flexible, and the resulting models can easily accommodate nonlinear relationships, make predictions about unseen data, be combined with simulations, incorporate different data types, and open the way to tools for testing generalization, such as cross-validation. For the data shown in Figure 1, a simple artificial neural network was used to map different profiles across multiple measures of short-term memory, working memory, fluid reasoning, vocabulary, and phonological awareness in a large group of struggling learners. Once the individual nodes of the network were trained to represent the different profiles within the data set, the locations of children with different diagnoses could be identified. If common diagnoses are predictive of the cognitive profiles learned by the network, then those children’s performance profiles should group together—but they did not. This highlights the potential utility of this approach to mapping the heterogeneity of a data set and exploring its composition. This is something largely untapped within the field to date.

### Beyond voxel-wise measures of brain structure and function

Just as methods that capture complexity in cognitive and behavioral data are needed, the same is true for neuroimaging. In theory, the methods outlined above can be used alongside neuroimaging, although in practice there are very few examples. SEM techniques could allow researchers to identify many-to-one mappings (e.g., Kievit et al., 2016; Kievit et al., 2014), allowing the possibility that a particular set of symptoms could be associated with multiple different neurobiological effects. And network science has enabled the subfield of brain “connectomics,” enabling researchers to demonstrate that apparently disparate neural effects could have very similar effects on brain organization, providing a meaningful endophenotype to bridge complex causal interrelations and shared surface-level profiles (e.g., Bathelt, Gathercole, Butterfield, the CALM Team, & Astle, 2018; Bathelt, Scerif, Nobre, & Astle, 2019).

## Conclusions

Core-deficit models long held promise as optimistic researchers aimed to interpret behavioral complexity via cognitive or neurological simplicity. However, as more and more of these attempts fall by the wayside, many researchers have come to question the validity of the principle of the core deficit. At the same time, increased pooling and sharing of data, as well as better diagnostic ascertainment, has improved our capacity to gather substantial samples for well-powered complex analyses. New technologies provide opportunities for creative, data-driven analysis of such samples, which can provide us with an empirical basis for the development of new theories.

We must embrace complexity within and between diagnostic boundaries in such theoretical models. In doing so, we will unlock our potential to understand the cross-cutting issues that frequently have the biggest impact on people’s lives rather than dwell on the narrow selection of domains that seem to be unique to a specific population. Importantly, a move away from the concept of “core” should also entail a movement away from the concept of “deficit”—there is no objective reason why a condition should be defined by its disadvantageous elements instead of its advantageous elements. Although the former may be needful of intervention, the latter may be essential to delivering that intervention. Examples of successful application of this principle come from formal evaluations—for example, technological supports for autistic children (Grynszpan, Weiss, Perez-Diaz, & Gal, 2014; Kasari et al., 2014)—but are also evident in practitioner-focused guides—for example, image-based rather than text-based learning materials for children with dyslexia (Mortimore & Dupree, 2008). In this way, the current direction of research in neurodevelopmental diversity is at a potential tipping point. The issues we outline above, and the recent developments we highlight, could have a beneficial impact not only on research innovation and knowledge generation but also on policy, practice, and societal understanding.

## Recommended Reading

Bathelt, J., Holmes, J., & Astle, D. E., on behalf of the Centre for Attention Learning and Memory (CALM) Team. (2018). (See References). A data-driven approach to exploring profiles of executive-functional difficulty and then testing whether they overlap with diagnostic status.

Bishop, D. V. (1997). (See References). A classic article that sowed the seeds for many of the questions we raise about developmental theory—required reading for anyone interested in developmental disorders.

Kievit, R. A., Hofman, A. D., & Nation, K. (2019). (See References). Excellent use of latent-change modeling to explore the relationship between different constructs over developmental time.

Mareva, S., the CALM Team, & Holmes, J. (2019). (See References). An excellent example of using a network analysis with a transdiagnostic sample to explore the interplay among different cognitive, communication, and behavioral characteristics.

Siugzdaite, R., Bathelt, J., Holmes, J., & Astle, D. E. (2020). (See References). Uses an artificial neural network to map between cognition and brain structure.

## Transparency

*Action Editor:* Randall W. Engle

*Editor:* Randall W. Engle
